# Comparison of phasing strategies for whole human genomes

**DOI:** 10.1371/journal.pgen.1007308

**Published:** 2018-04-05

**Authors:** Yongwook Choi, Agnes P. Chan, Ewen Kirkness, Amalio Telenti, Nicholas J. Schork

**Affiliations:** 1 J. Craig Venter Institute, Rockville, Maryland, United States of America; 2 Human Longevity, Inc., San Diego, California, United States of America; 3 J. Craig Venter Institute, La Jolla, California, United States of America; 4 University of California San Diego, La Jolla, California, United States of America; 5 The Translational Genomics Research Institute (TGen), Phoenix, Arizona, United States of America; University of Oxford, UNITED KINGDOM

## Abstract

Humans are a diploid species that inherit one set of chromosomes paternally and one homologous set of chromosomes maternally. Unfortunately, most human sequencing initiatives ignore this fact in that they do not directly delineate the nucleotide content of the maternal and paternal copies of the 23 chromosomes individuals possess (i.e., they do not ‘phase’ the genome) often because of the costs and complexities of doing so. We compared 11 different widely-used approaches to phasing human genomes using the publicly available ‘Genome-In-A-Bottle’ (GIAB) phased version of the NA12878 genome as a gold standard. The phasing strategies we compared included laboratory-based assays that prepare DNA in unique ways to facilitate phasing as well as purely computational approaches that seek to reconstruct phase information from general sequencing reads and constructs or population-level haplotype frequency information obtained through a reference panel of haplotypes. To assess the performance of the 11 approaches, we used metrics that included, among others, switch error rates, haplotype block lengths, the proportion of fully phase-resolved genes, phasing accuracy and yield between pairs of SNVs. Our comparisons suggest that a hybrid or combined approach that leverages: 1. population-based phasing using the SHAPEIT software suite, 2. either genome-wide sequencing read data or parental genotypes, and 3. a large reference panel of variant and haplotype frequencies, provides a fast and efficient way to produce highly accurate phase-resolved individual human genomes. We found that for population-based approaches, phasing performance is enhanced with the addition of genome-wide read data; e.g., whole genome shotgun and/or RNA sequencing reads. Further, we found that the inclusion of parental genotype data within a population-based phasing strategy can provide as much as a ten-fold reduction in phasing errors. We also considered a majority voting scheme for the construction of a consensus haplotype combining multiple predictions for enhanced performance and site coverage. Finally, we also identified DNA sequence signatures associated with the genomic regions harboring phasing switch errors, which included regions of low polymorphism or SNV density.

## Author summary

Humans are a diploid species that inherit one set of chromosomes paternally and one set of chromosomes maternally. Separating the nucleotide content of the maternally and paternally-derived chromosomes for an individual, i.e., ‘phasing’ that individual’s genome, is not trivial with today’s sequencing technologies. This is in part due to the fact that most available sequencing technologies generate short sequencing reads that make it hard to assemble individual homologous chromosome pairs. Phase information can be crucial for putting into context the likely functional consequences of DNA sequence variants as well as certain evolutionary and population genetics phenomena. In order to assess the reliability of current sequencing-based phasing strategies, we compared 11 different approaches using a public domain reference genome as a test case. These phasing strategies included laboratory-based experimental techniques as well as purely computational approaches. Importantly, our comparisons show that a hybrid or combined approach that leverages population-based phasing via the SHAPEIT software suite works well and can be improved with the addition of genome-wide sequence read or parental genotype data.

## Introduction

Whole genome sequencing (WGS) of individual human genomes has been made possible by rapid advances in DNA sequencing technology (reviewed in [1]). In fact, an initial draft of an individual’s genome, which results in spelling out over 3.2 billion base pairs, now costs approximately $1,000. The exploitation of WGS in association and clinical studies has led to some remarkable successes in the identification of disease-causing, overtly pathogenic variants [2] as well as the use of these variants in diagnostic, prognostic and pharmacogenetic assessment settings [3]. Unfortunately, due to inherent limitations in current sequencing technologies, such as short DNA sequence read lengths, a number of issues remain. For example, sequencing strategies are not error-free, making it possible that some nucleotides in an individual genome are erroneously assigned. This error varies greatly among sequencing technologies [1,4]. In addition, although virtually all sequencing technologies can capture simple single nucleotide variants (SNVs), the identification of some forms of structural variation (e.g., moderately sized insertion/deletion variants (indels) and complex copy number variants (CNVs)) have proven difficult to identify in a straightforward manner [5,6]. Steady improvements in sequencing laboratory protocols and computational workflows are beginning to overcome these issues [6], but they also contribute to another, perhaps more fundamental issue surrounding the fact that humans are diploid and possess two ‘genomes:’ one inherited maternally and one paternally as a set of 23 chromosome pairs. Understanding how *cis* (i.e., near each other on the same chromosome) and *trans* (far from each other or on opposite chromosomes) combinations of variants impact phenotypic expression is likely to be of crucial importance in human biology. This activity depends critically on having phase information, i.e., knowing the unique nucleotide content of each of the two chromosomes making up the 23 pairs.

Most genome sequencing strategies derive DNA sequence for the 23 chromosome pairs simultaneously from a pool of maternally and paternally-derived DNA (e.g., from blood samples) and hence do not segregate the nucleotide content of each copy of a homologous chromosome pair. Further, most strategies seek to identify nucleotides at each position of a genome and, by comparing nucleotides of the sequenced genome to the sequence of nucleotides for a vetted reference genome, identify SNVs and possibly other forms of variation. In general, each SNV position is then recorded as having either one copy of a non-reference nucleotide and one copy of a reference nucleotide (i.e., ‘heterozygous’), two copies of a non-reference nucleotide (i.e., ‘homozygous non-reference’), two copies of the ‘reference’ nucleotide (i.e., ‘homozygous reference’), or, rarely, two different non-reference nucleotides. Note that phase information is really only relevant for heterozygous loci (i.e., where there is a difference in the nucleotide content of homologous chromosome pair). Obtaining phase (or haplotype) information is often non-trivial, as reviewed by us and other research groups [7–9].

As noted, there are a number of very important genetically-mediated phenomena for which characterization depends critically on phase information. For example, the phenomenon of compound heterozygosity, in which two different mutations, one impacting the function of paternally-inherited homologous copy of a gene and the other impacting the function of the maternally-inherited homologous copy of that gene, can only be distinguished from situations in which the two mutations reside on the same copy of the gene by having phase information [7,10]. This is particularly important for the highly polymorphic human leukocyte antigen (HLA) region of the genome. In addition, phase information is also required in dissecting many evolutionary and population genetics phenomena, such as haplotype frequency differences across populations and linkage disequilibrium (LD) patterns. For example, LD patterns around specific mutations are useful for determining the age of those mutations as well as imputing variants to individuals that have not been sequenced or genotyped at the relevant position in the sequence [11].

A number of strategies have been, or are being, developed to generate phase information [7,8]. Some of these strategies involve elaborate laboratory protocols, others rely on *de novo* assembly computational methods, and yet others make use of population haplotype frequency information. Sequencing read lengths and the depth (i.e., coverage) of sequencing for a genome play large roles in the ability to phase a genome. By far the most straightforward, most often-used, and most-intuitive strategy for obtaining phase information from genome sequencing involves sequencing the parents (and/or other relatives) of an individual whose genome is to be phased. This way, Mendel’s laws can be used to essentially observe which variants were inherited from each of the parents. Unfortunately, even with parental sequence information there are problems that can plague obtaining phase information. For example, if the mother, father, and offspring are all heterozygous at a locus, the parental origin of the variants in the offspring cannot be determined, and the phase information for variants at that locus will remain ambiguous.

We compared a variety of methods for phasing entire human genomes leveraging the publicly available genome of a European (i.e., ‘CEU,’ per community conventions) female individual NA12878. The NA12878 ‘Genome-In-A-Bottle (GIAB)’ reference was constructed based on multiple-platform arbitrated genotype [12] and haplotype information by multiple research groups at Illumina, Inc. and Real Time Genomics, Inc. using a suite of techniques and sequence information available from the relatives of NA12878 [13,14]. To actually compare the performance of the different phasing strategies, we considered laboratory-based phasing methods by analyzing previously published datasets (i.e., we used what was published, since we did not have access to the raw material and laboratory protocols to recreate them), as well as computational phasing strategies that we were able to put together based on available software. We recognize that the use of publicly available genomes could be biased since the research groups generating the data may have used many protocols and workflows that are not necessarily available to the public, but we argue that our analyses of those public domain phased genomes provides an ‘upper bound’ (i.e., best case scenario) on their accuracy. We benchmarked the accuracy of the laboratory and computational phasing approaches using a variety of metrics including switch error rates, haplotype block lengths, and the proportion of fully phase-resolved genes. Our results suggest that hybrid computational approaches to phasing a single genome are accurate and cost-effective, and exhibit a performance that nearly matches or surpasses laboratory-based approaches.

## Methods

### Source of data

#### Gold standard variants and haplotypes for the NA12878 genome

We obtained genome-wide variant data and phase-resolved haplotype information for the reference individual NA12878 from the GIAB v0.2 VCF file, available from the National Institute of Standards and Technology (NIST) Genome-In-A-Bottle (GIAB) consortium FTP site [15]. A total of 1,697,789 phase-resolved heterozygous SNVs on 22 autosomes were extracted from the GIAB v0.2 VCF file and used as the gold standard in our phasing performance analyses. For the X chromosome, 55,108 phase-resolved heterozygous SNVs were extracted and used for a separate analysis since the phased data for the X chromosome were not available for all phasing approaches we considered and special independent processing steps are required for some approaches to evaluate the X chromosome. The GIAB v0.2 VCF file included the NIST-GIAB multiple-platform arbitrated genotype calls [12] integrated with haplotype calls from the Illumina Platinum and Real Time Genomics (RTG) independent phasing efforts. These variant calls incorporated data from multiple sequencing platforms and leverage the genotype and sequence data available on NA12878’s extended pedigree [13,14]. We compared different phasing strategies by obtaining genotype data, whole genome shotgun DNA reads, RNA-Seq reads available for NA12878, as well as parental genotype, applying different phasing methods to these data, and then comparing the accuracy of each phasing method against the GIAB v0.2 VCF phase-resolved variant calls.

#### NA12878 sequence data and parental genotype

All sequence data for NA12878 were obtained from the NCBI internet resource (Sequence Read Archive), including: PacBio reads (SRR1947646, SRR1950266-SRR1950290; 50X coverage [16]), Illumina paired-end DNA reads (SRR2052404, SRR2052414-SRR2052424; 45X coverage), and Illumina paired-end RNA reads (SRR1153470, SRR1258218, ERR356372; 300 million PE reads). The genotype data for the parents (NA12891 and NA12892) were obtained from Illumina BaseSpace [17].

#### In-house variant calls for NA12878

We generated an in-house genome-wide variant dataset for NA12878 using standard bioinformatics methods to benchmark and compare various phasing strategies. The in-house set of NA12878 variant calls was generated with the reference human genome GRCh37 coordinates with the Illumina paired-end DNA reads (45X coverage, 148 bp), using BWA-MEM [18] for read mapping and GATK [19] for variant calling.

### Phasing methods compared

We compared 11 different approaches for phasing the NA12878 genome. The approaches considered are briefly described below with references provided where appropriate. We also refer the reader to websites associated with the commercial groups whose products enable some of these approaches. The descriptions below consider the laboratory-based methods first, followed by computational methods.

1. 10X Genomics (10X). This commercially available approach uses micro-droplet-based dilution methods to randomly compartmentalize DNA molecules into partitions carrying ultra-low genome equivalents (~0.002 genome per partition). The 10X strategy uses a relatively higher number of distinct barcodes than other laboratory-based phasing methods (i.e., ~100,000 in 2015 when the data were made available) and a minimal amount of haploid genome equivalents per partition. The approach reduces the chance of DNA molecules in a given partition originating from the same genomic loci [20]. Data for the NA12878 haplotypes determined by 10X Genomics method were downloaded from the GIAB FTP site [21].
2. Contiguity Preserving Transposon (CPT-seq). This strategy uses a proprietary two-tiered dilution-based method developed by Illumina, Inc. [22]. High molecular weight DNA is first tagged with 96 different transposon tags and pooled. The pooled samples are further indexed with 96 sets of PCR primers to produce roughly 10,000 virtual compartments. All compartments are then mixed, sequenced as a single sample, de-multiplexed, and mapped to the reference genome to determine long range linkage information. Data for the CPT phased NA12878 were obtained from Illumina (Steemers et al., Illumina, Inc. personal communication).
3. Fosmid-pool-based Phasing Strategy. This method [23] uses 40-kb haploid DNA segments from fosmid pool-based NGS. Reads were assembled using the ReFHap software [24]. Assembled haplotype data were downloaded from the Max Planck institute website [25]. For comparison with the NA12878 GIAB gold standard and other phasing approaches, genomic coordinates from NA12878 fosmid study were converted from NCBI36 (hg18) to GRCh37 (hg19) using the liftOver tool [26].
4. Moleculo. The Moleculo approach uses a proprietary dilution-based method developed by Illumina, Inc. for phasing genomes [27]. A complexity reduction approach is used to obtain sequence information from pools of roughly 10 kb genomic fragments that belong to different parts of the genome. Data for the phased Moleculo NA12878 genome were downloaded from the Illumina BaseSpace website (Steemers, Illumina, Inc. personal communication) [28].
5. Beagle (version 4.0 r1399). This computational phasing algorithm represents one of the early HMM-based approaches. It essentially samples different possible haplotype arrangements to find the most likely haplotype pair for an individual conditional on the individual’s genotypes [8]. The algorithm scales quadratically with input data. Beagle was one of the tools used to estimate haplotypes for the 1000GP phase 1 array-based genotype data [29].
6. Eagle2 (version v2.3.1). This is a recent reference-based computational phasing algorithm that efficiently leverages information from large external reference panels. The computational efficiency of this approach is realized through a data structure based on positional Burrows-Wheeler transform and a rapid search algorithm that explores only the most relevant paths through a hidden Markov model (HMM) [30].
7. SHAPEIT (version v2.r837, also referred to as SHAPEIT2). This computational phasing algorithm uses an HMM-based approach to estimate an individual’s haplotypes based on genotype data from a population [31]. Given an individual’s genotype data, all possible haplotypes are represented in a graphical model. Population-based constraints are then applied to find a pair of paths through the graph to determine the haplotype of the individual. The approach is fast and efficient as the algorithm has a linear complexity with the number of SNVs and possible haplotype space. SHAPEIT has been used to estimate haplotypes for the 1000 Genomes Project (1000GP) [32] and we used the available genotype data from this effort for our analyses. In addition, the SHAPEIT algorithm can be further supplemented with sequence read data or parental genotype for improved performance, as described below.
8. SHAPEIT with WGS reads and/or RNA-Seq Reads. In addition to using the NA12878 genotype information available in the public domain, we applied the SHAPEIT program with the addition of WGS reads (Illumina or PacBio) and/or RNA-Seq reads data after mapping.
9. SHAPEIT with Parental Genotype Data. In addition to the individual’s genotype to be phased, the parental genotype data were supplied and phased together using a haplotype reference panel.
10. HapCUT (version 0.7 updated on 9/11/2013) with the WGS Reads. This computational phasing algorithm addresses haplotype phasing as a haplotype assembly problem using DNA sequence fragments rather than population genotypes. HapCUT reconstructs the haplotypes of an individual’s genome based on overlapping sequence fragments that carry two or more variant sites. Sufficient sequencing depth and large overlaps are the major factors for reconstructing long haplotypes with the HapCUT program. The HapCUT algorithm uses a graph-based approach to represent overlaps among sequence fragments and tries to minimize an error score of the reconstructed haplotypes by iteratively computing high-scoring cuts in the graph [33]. We used the HapCUT algorithm with the Illumina paired-end or PacBio long WGS DNA reads.
11. Majority Vote Phasing. We developed a simple majority vote approach to combine multiple haplotype predictions described above to test for any enhanced phase resolution that could arise from a consensus analysis. In this approach, heterozygous SNV sites are incrementally phased with respect to previously phase-determined upstream sites using predicted phase information from multiple methods. For a given site to be phased, the upstream site, which may be different for each method, also needs to be considered. There are 2 possibilities for the phase at the target site with respect to the upstream site. Each phasing method casts a vote to the 2 possibilities, and the combined phase is simply determined by majority voting. The same procedure is repeated on each chromosome for all heterozygous SNVs.

### Haplotype reference panels

Population-based phasing, such as those strategies implemented in Beagle, Eagle2, and SHAPEIT software packages, makes use of a haplotype reference panel for haplotype frequency information. For this study, the 1000GP phase 3 reference panel dataset was used and contains 2,503 individuals excluding individual NA12878. We also used the Haplotype Reference Consortium (HRC) panel dataset which contains 22,690 individuals excluding individual NA12878 [34]. The HRC reference panel included WGS datasets from over 20 studies to develop a large combined haplotype reference panel, currently with predominantly European ancestry. The HRC reference panel also included the 1000GP individuals. The 1000GP reference panel for Beagle and SHAPEIT runs were obtained from the corresponding project web sites of the phasing tools [35,36]. The HRC reference panel was obtained from the European Genome-Phenome Archive (EGA) (Dataset ID: EGAD00001002729) [37].

### Metrics for phasing performance

We considered five different metrics to assess phasing performance. All have been used in previous research to assess how well individual genomes can be phased. Each of these metrics is described briefly below. The datasets and scripts used for comparing the phasing methods is available at https://github.com/ywchoi/phasing.

#### Percentage of phased single nucleotide variants (SNVs)

We compared the results of each of the phasing strategies with the gold standard GIAB genome (which harbored ~1.7 million heterozygous SNVs) and determined the percentage of SNVs that could be phase-resolved into a haplotype by the strategies we considered.

#### Switch error rate (SER)

Phasing accuracy is typically measured by counting the number of ‘switches’ between known maternal and paternal haplotypes that should not occur if individual maternal and paternal chromosomal nucleotide sequence content has been accurately characterized. If an inconsistency is identified, then it is called a ‘switch error.’ These switch errors manifest themselves as induced and false recombination events in the inferred haplotypes compared with the true haplotypes. To identify switch errors, the phase of each site is compared with upstream neighboring phased sites. The switch error rate (SER) is defined as the number of switch errors divided by the number of opportunities for switch errors. Switch errors were further classified into three categories: long, point, and undetermined. A long switch appears as a large-scale pseudo recombination event; that is, there are no other switches in the local neighborhood around the long switch (e.g., no other switches within three consecutive heterozygous sites). On the contrary, a small-scale switch error appearing as two neighboring switch errors is considered as a point switch (e.g., two switches within three consecutive heterozygous sites, with the pair of switches counted as a point switch). The remaining switches are considered undetermined (e.g., only two sites phased in a small phasing block, so the switch error could not be classified into long or point).

#### Haplotype block length

As introduced by Duitama et al. [23], we used Quality Adjusted N50 (QAN50) haplotype block length to characterize the completeness and quality of phased haplotypes. Phasing approaches typically produce chromosomes that are broken into one or more phasing blocks. A phasing block is a region that is declared completely phased by the approach but potentially contains switch errors. Individual phasing blocks were further broken up, at every error site, into multiple sub-blocks. Then each sub-block length was first computed as the number of nucleotides from the first to last sites. The sub-block length was then adjusted by multiplying by the proportion of phased sites within the sub-block. These lengths were referred to as the ‘quality adjusted (QA) haplotype block lengths.’ QAN50 is defined as the largest QA haplotype block length such that 50% of all heterozygous sites represented in the gold standard are contained in haplotype blocks of quality adjusted length at least QAN50.

#### Pairwise SNVs phasing accuracy and phasing yield

In addition to the overall switch error rate, to assess phasing performance with respect to the distance between a pair of SNVs, pairwise phasing yield and accuracy were measured as a function of the distance between the SNV pairs as described previously [8,22]. Ultimately, phasing accuracy was assessed as the probability that a pair of SNVs in the same phasing block was phased correctly as a function of the distance between the pair. In addition, phasing yield represents the probability that a pair of SNVs are phased in the same phasing block as a function of the distance between the pair.

#### Percentage of genes fully phased

Out of the 19,430 protein coding genes on the autosomal chromosomes (i.e., 1 to 22) annotated in the Ensembl gene annotation resource (Release 75), a total of 12,814 genes carried more than one heterozygous variant in the NA12878 genome. This set of 12,814 autosomal genes was used as a reference baseline to compute the percentage of fully phased genes by the various phasing strategies compared in this study. A gene is considered fully phased in a phasing approach if all heterozygous SNVs on the gene are correctly phased with respect to the GIAB gold standard.

#### SER as a function of haplotype diversity

Haplotype diversities were estimated using the 1000GP reference panel. Given a set of haplotypes from the 1000GP population within a non-overlapping window of size 1000bp on a chromosome, the haplotype diversity is defined as the probability of having different haplotypes when two haplotypes are randomly drawn [38]. SERs were computed separately for each non-overlapping interval of haplotype diversity (i.e., from 0 to 0.05, from 0.05 to 0.1, etc., and ultimately from 0.95 and 1).

#### Shared switch error sites

For every pair of phasing approaches, we computed the number of switch errors that occurred at the same position. To measure the similarity of the two approaches, the Jaccard index [39] was calculated as the number of common errors divided by the size of the union of the total errors sites.

## Results

### Comparison of phasing strategies

A total of 11 phasing approaches were compared based on the NIST-GIAB reference genome NA12878. Laboratory-based experimental phasing previously reported from 10X Genomics, CPT-seq, fosmid sequencing, and Moleculo were collected from the literature and public datasets. Computational phasing results were generated in-house for the NA12878 genome using Beagle [32], Eagle2 [30], SHAPEIT [31], and HapCUT [33] with one or more combinations of the following as input data for the relevant computations: the haplotype reference panels from the 1000 Genomes Project (1000GP) with 2.5k individuals or the haplotype references from the Haplotype Reference Consortium (HRC) with 23k individuals, conventional WGS reads generated from the Illumina or PacBio sequencing platforms, RNA-Seq reads, or parental genotype.

#### Laboratory-based phasing approaches

**Fig 1:**
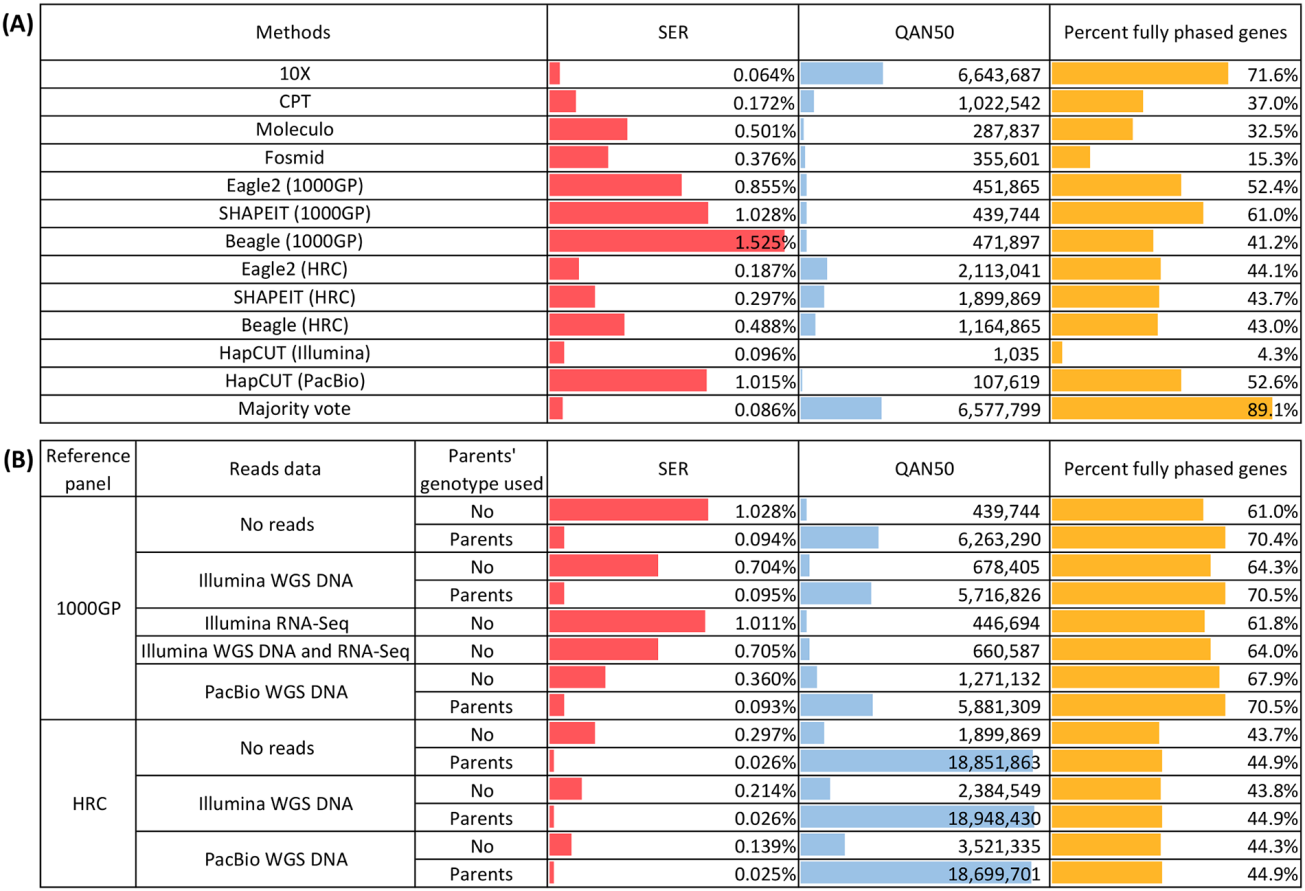
Summary of phasing performance as shown by switch error rates, Quality Adjusted N50 (QAN50), and the percentage of fully phased genes. (A) All phasing methods tested, and (B) SHAPEIT phasing making use of different combinations of reference panels, WGS/RNA read data, and parental genotype.

Among the laboratory-based phasing methods analyzed, conventional fosmid sequencing and the genome reduction dilution-based Moleculo approach produced haplotype blocks of moderate QAN50 lengths, ~0.4 and ~0.3 Mb, respectively. More recently developed dilution-based approaches, such as CPT-seq and 10X Genomics, further reduce the required haploid genome equivalents in individual partitions using various laboratory constructs that dramatically increase the range of the genome that could be ‘barcoded’–and hence have the potential to be phased—by orders of magnitude, effectively enabling ultra-high-throughput processing and pooling. The CPT-seq and 10X Genomics approaches produced haplotype blocks surpassing mega-base QAN50 lengths (1 and 7 Mb, respectively) with low switch error rates (SER) (0.17 and 0.06%, respectively) (Fig 1A, Table 1 and S1 Fig).

**Table 1:** Performance summary across experimental-, population-, read-based, and majority vote phasing approaches.

Methods		10X	CPT	Moleculo	Fosmid	Eagle2 (1000GP)	SHAPEIT (1000GP)	Beagle (1000GP)	Eagle2 (HRC)	SHAPEIT (HRC)	Beagle (HRC)	HapCUT (Illumina)	HapCUT (PacBio)	Consensus by majority vote
# phasing blocks		329	2,684	8,417	12,145	22	22	22	22	22	22	319,507	22,187	22
# phased SNVs		1,662,926	1,558,391	1,568,032	1,342,990	1,660,410	1,675,084	1,644,275	1,596,504	1,596,504	1,596,504	1,417,643	1,686,245	1,690,767
% phased SNPs (among golden set)		97.9%	91.8%	92.4%	79.1%	97.8%	98.7%	96.8%	94.0%	94.0%	94.0%	83.5%	99.3%	99.6%
QAN50		6,643,687	1,022,542	287,837	355,601	451,865	439,744	471,897	2,113,041	1,899,869	1,164,865	1,035	107,619	6,577,799
# SNPs used for switch error measurement		1,662,597	1,555,707	1,559,447	1,330,845	1,660,388	1,675,062	1,644,253	1,596,482	1,596,482	1,596,482	1,098,136	1,664,058	1,690,745
# switch errors		1,058	2,677	7,807	5,007	14,199	17,214	25,077	2,991	4,736	7,784	1,050	16,889	1,452
SER		0.064%	0.172%	0.501%	0.376%	0.855%	1.028%	1.525%	0.187%	0.297%	0.488%	0.096%	1.015%	0.086%
# switch errors (total = long + 2*point + undetermined)	Long	11	519	1,891	883	3,363	2,694	7,334	1,181	1,293	2,700	175	8,379	387
	Point	515	935	2,492	1,867	5,416	7,257	8,871	904	1,720	2,541	91	3,160	530
	Undetermined	17	288	932	390	4	6	1	2	3	2	693	2,190	5
% genes fully phased	Among 12,814 genes with more than 1 heterozygous SNVs	71.6%	37.0%	32.5%	15.3%	52.4%	61.0%	41.2%	44.1%	43.7%	43.0%	4.3%	52.6%	89.1%
% genes phased at least 90% SNVs without switch errors		93.0%	70.9%	61.2%	24.9%	75.1%	74.9%	68.4%	78.7%	77.3%	75.1%	5.7%	62.3%	96.3%
% switch errors in repeat regions		54.3%	49.9%	49.3%	45.4%	50.7%	49.5%	52.7%	49.5%	48.3%	49.9%	58.1%	52.6%	62.8%

#### Computational population-based approaches

For population-based computational phasing approaches, predefined haplotypes from a reference panel of individuals are leveraged to predict the haplotypes for the NA12878 genome. With the 1000GP reference panel, Beagle, Eagle2, and SHAPEIT, produced modest size haplotype blocks with QAN50 between 0.4 and 0.5 Mb, and SER of 1.53%, 0.86%, 1.03%, respectively. Using the HRC reference panel, which consists of about 10 times more individuals than the 1000GP panel, Beagle, Eagle2 and SHAPEIT produced increased haplotype block QAN50 sizes by about 2 to 5 times to ~1 to 2 Mb and the SER was reduced to 0.49%, 0.19% and 0.30%, respectively (Fig 1A and 1B, Table 1 and S1 Fig).

#### Computational read-based approach

Read-based phasing uses conventional whole genome sequencing (WGS) reads as input data to extract physical linkage information and then reconstructs haplotypes based on overlapping sequence fragments carrying two or more variant sites. HapCUT is a read-based phasing method and produced relatively short haplotype block sizes at 1 kb in our study, and with low errors and an SER of 0.10% when using Illumina WGS reads. Longer haplotype blocks in the range of ~0.1 Mb with an SER of 1.01% were produced when using PacBio WGS reads instead (Fig 1A and 1B, Table 1 and S1 Fig). HapCUT like any other read-based approaches does not require reference haplotypes and therefore would have the advantage of being able to resolve variants specific to the individual in question.

#### Hybrid population and read-based approaches

In addition to the statistical approach based purely on haplotype frequency data from a reference panel of genomes, the SHAPEIT algorithm can also incorporate physical linkage information provided by conventional WGS reads. Phase-informative reads (PIRs) were extracted from the initial set of NA12878 Illumina or PacBio WGS reads providing physical linkage information between neighboring variants. The inclusion of Illumina (45X) or PacBio (50X) WGS reads to SHAPEIT phasing with the HRC panel improved the QAN50 lengths from ~1.94 Mb for SHAPEIT alone to ~2.4 Mb with the inclusion of Illumina reads or ~3.5 Mb with PacBio reads (Fig 1B, Table 2, and S1 Fig). The SER was reduced from 0.30% for SHAPEIT alone to 0.21% after including Illumina WGS reads and 0.14% with PacBio WGS reads. Importantly, the hybrid approach of SHAPEIT phasing with the inclusion of WGS data improved QAN50 length by up to 85% and reduce SER by up to 53%.

**Table 2:** Performance summary of population-based phasing approaches supplemented with sequence reads and/or parental genotype information.

SHAPEIT approaches	Reference panel	1000GP								HRC					
	Reads data	No reads		Illumina WGS DNA		Illumina RNA-Seq	Illumina WGS DNA and RNA-Seq	PacBio WGS DNA		No reads		Illumina WGS DNA		PacBio WGS DNA	
	Parents’ genotype used	No	Parents	No	Parents	No	No	No	Parents	No	Parents	No	Parents	No	Parents
# phasing blocks		22	22	22	22	22	22	22	22	22	22	22	22	22	22
# phased SNVs		1,675,084	1,674,475	1,675,084	1,674,475	1,675,084	1,675,084	1,675,084	1,674,475	1,596,504	1,596,288	1,596,504	1,596,288	1,596,504	1,596,288
% phased SNPs (among golden set)		98.7%	98.6%	98.7%	98.6%	98.7%	98.7%	98.7%	98.6%	94.0%	94.0%	94.0%	94.0%	94.0%	94.0%
QAN50		439,744	6,263,290	678,405	5,716,826	446,694	660,587	1,271,132	5,881,309	1,899,869	18,851,863	2,384,549	18,948,430	3,521,335	18,699,701
# SNPs used for switch error measurement		1,675,062	1,674,453	1,675,062	1,674,453	1,675,062	1,675,062	1,675,062	1,674,453	1,596,482	1,596,266	1,596,482	1,596,266	1,596,482	1,596,266
# switch errors		17,214	1,568	11,795	1,587	16,934	11,815	6,036	1,564	4,736	414	3,414	411	2,218	403
SER		1.028%	0.094%	0.704%	0.095%	1.011%	0.705%	0.360%	0.093%	0.297%	0.026%	0.214%	0.026%	0.139%	0.025%
# switch errors (total = long + 2*point + undetermined)	Long	2,694	75	2,370	84	2,680	2,444	1,663	75	1,293	21	1,103	19	754	19
	Point	7,257	746	4,710	751	7,123	4,683	2,183	744	1,720	196	1,153	196	730	192
	Undetermined	6	1	5	1	8	5	7	1	3	1	5	-	4	-
% genes fully phased	Among 12,814 genes with more than 1 heterozygous SNVs	61.0%	70.4%	64.3%	70.5%	61.8%	64.0%	67.9%	70.5%	43.7%	44.9%	43.8%	44.9%	44.3%	44.9%
% genes phased at least 90% SNVs without switch errors		74.9%	92.9%	80.1%	92.9%	75.9%	79.9%	86.8%	93.0%	77.3%	82.8%	78.4%	82.8%	80.2%	82.8%
% switch errors in repeat regions		49.5%	56.4%	49.4%	56.2%	49.7%	49.3%	50.0%	56.9%	48.3%	54.1%	48.6%	51.6%	50.6%	54.3%

We also explored the added values of RNA-Seq reads for this hybrid approach. The assumption is that RNA-Seq reads would provide the added advantages of spanning adjacent exon sequences and linking long range variants. Phase-informative reads (PIRs) were extracted from an NA12878 RNA-Seq run (300 million PE reads) for SHAPEIT phasing with the 1000GP reference panel. The QAN50 length was 0.44 Mb for SHAPEIT alone, 0.45 Mb with added RNA reads, and 0.68 Mb with added Illumina WGS reads. Considering the proportion of correctly phased genes with ≧90% sites phased, the performance was 74.9% (9,594 genes) for SHAPEIT alone, 75.9% (9,721 genes) for added RNA reads, and 80.1% (10,262 genes) for added Illumina WGS reads. Overall, we observed no appreciable differences in terms of haplotype block length improvements with the addition of RNA-Seq reads to SHAPEIT phasing. Considering the total number of correctly phased genes, there was a gain of ~100 genes with the addition of RNA reads to SHAPEIT phasing.

#### Hybrid population and trio-genotype approach

In rare genetic disorders parent offspring trios are often sequenced altogether, for example for screening recessively inherited *de novo* mutations. We considered another hybrid phasing approach by making use of a reference panel of haplotypes and the parents’ genotype data for SHAPEIT phasing (i.e., we included genotype data from the parents of NA12878 (i.e., NA12891 and NA12892)). A SHAPEIT phasing run was performed using the HRC panel as background with the inclusion of the NA12878 parental genotypes. This run produced 19 Mb haplotype blocks, compared to only 1.9 Mb for SHAPEIT alone, or 3.5 Mb for SHAPEIT with PacBio reads (Fig 1B, Table 2, and S1 Fig). As for the SER, the inclusion of parental genotype reduced overall errors from 0.30% for SHAPEIT alone to 0.03%. The inclusion of parental genotype also corrected substantially more long switches compared to point switches. Importantly, the addition of parental genotype data drastically improved both the haplotype block length and SER 10 times, which was also the longest haplotype blocks and lowest SER across all phasing approaches tested in this study.

Family trios are traditionally phased by transmission following the application of Mendel’s laws, but for sites that are heterozygous across all members they are non-resolvable by Mendel’s laws alone. To estimate the proportion of sites that are non-resolvable in the NA12878 family trios, we scanned the genotypes of the trio and summed all triply heterozygous sites (i.e. sites that are heterozygous for all 3 individuals). Among 2.5 million heterozygous SNV sites in NA12878, a total of 360k sites were found to be heterozygous in both parents, and an additional 464k sites were found to have no variant called in both parents (S1 Table). Thus, a total of 824k heterozygous SNV sites of the NA12878 individual are non-resolvable by inheritance. In other words, at most 67% of the heterozygous SNV sites in the NA12878 individual can be resolved relying on the conventional parent offspring transmission alone. This further highlights the important favorable utility of parental genotype data in the context of enhancing population-based phasing accuracy as demonstrated above.

#### Phasing accuracy for rare variants

**Fig 2:**
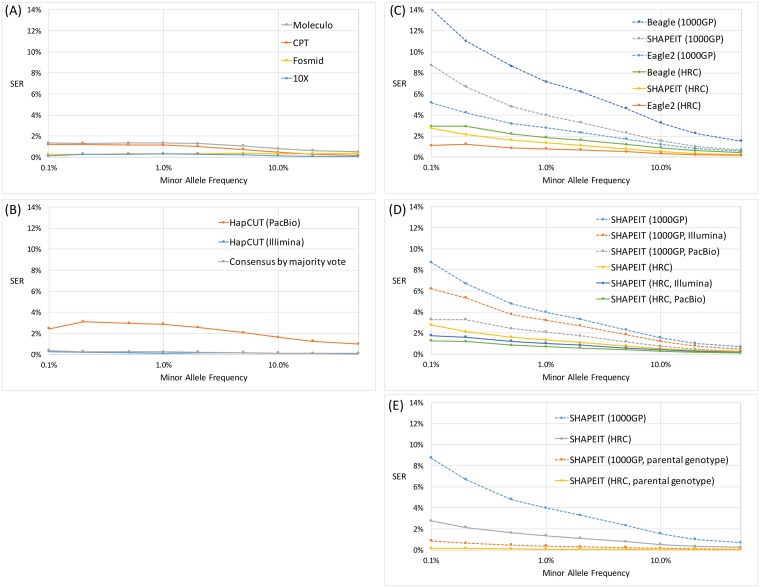
Switch error rates across phasing strategies as a function of minor allele frequency. (A) Laboratory-based phasing, (B) Read-based and majority voting, (C) Population-based phasing, (D) Hybrid population and read-based, and (E) Hybrid population and familial data from parental genotype.

Because statistical phasing of rare variants is generally more difficult for population-based phasing strategies given the need for accurate variant frequency and linkage disequilibrium (LD), we investigated the accuracy of phased-resolved rare variants when the minor allele frequency (MAF) was below 1%. SER was computed at each given MAF cutoff from 0.1, 0.2, 0.5 to 1% and above (Fig 2). As expected, for laboratory-based methods that solely rely on efficient partitioning of haploid DNA fragments and physical linkage of sites rather than population information, such as 10X, fosmid, CPT, and Moleculo, the SER remain largely stable at 0.1, 0.3, 1.2, and 1.3% respectively, even when the MAF is less than 0.1% (Fig 2A). In addition, the read-based approach HapCUT and our majority vote approach (described in the next section) also showed stable SERs at low MAF sites (Fig 2B). Across population-based methods, using either the 1000GP or the HRC reference panel, the SER in general increased about 2-fold as the MAF cutoff decreased from 1 to 0.1%. Switching from the 1000GP to the HRC reference panel, which is 10 times larger and also inclusive of the 1000GP panel, greatly reduced the SER at low MAFs. When we made use of the HRC panel, at MAF cutoff of 1 and 0.1% the SER for Eagle2 was 1.1 and 0.8% and those for SHAPEIT was 2.8 and 1.4%, respectively (Fig 2C). The addition of Illumina or PacBio WGS reads to the SHAPEIT phasing run also reduced SER for rare variants with MAF less than 1%, with performance approaching laboratory-based methods (Fig 2D). Importantly, SHAPEIT phasing supplemented with the parental genotype (as described above) also outperformed laboratory-based methods with SER improvement not only among common variants but also rare variants with MAF less than 1% (Fig 2E).

#### A majority vote approach to enhance phasing performance

To investigate whether combining haplotype prediction from multiple methods can boost phasing performance, we developed a majority vote approach. Different phasing methods likely cover different, but overlapping, subsets of SNV sites for any one genome suggesting that combining them may yield more accurate phasing. In our majority voting approach, heterozygous SNV sites are incrementally phased with respect to already phased upstream sites using all available predicted phase information from multiple methods. We combined the predicted haplotypes from 8 phasing methods including 4 lab-based and 4 computational methods. The phasing methods considered were 10X, CPT, fosmid, Moleculo, Beagle (HRC), SHAPEIT (HRC), Eagle2 (HRC), and HapCUT (Illumina), which produced SERs in the range of 0.06% (10X) to 0.50% (Moleculo), the proportion of phased SNVs between 79.1% (fosmid) and 97.9% (10X), and fully phased genes between 4.3% (HapCUT/Illumina) and 71.6% (10X) (S2 Fig). The new combined SER obtained from majority voting was 0.09%, with 99.6% SNVs phased, and 89.1% fully phased genes. In summary, using the majority voting approach to combine predicted haplotypes from multiple methods can boost phasing performance. As an example, our majority vote approach was able to fully phase-resolve 11,416 out of 12,814 phasable genes (i.e. genes having 2 or more heterozygous SNVs), compared to 9,174 genes by the best available individual phasing approach.

#### Phasing accuracy and yield across pairs of SNVs

**Fig 3:**
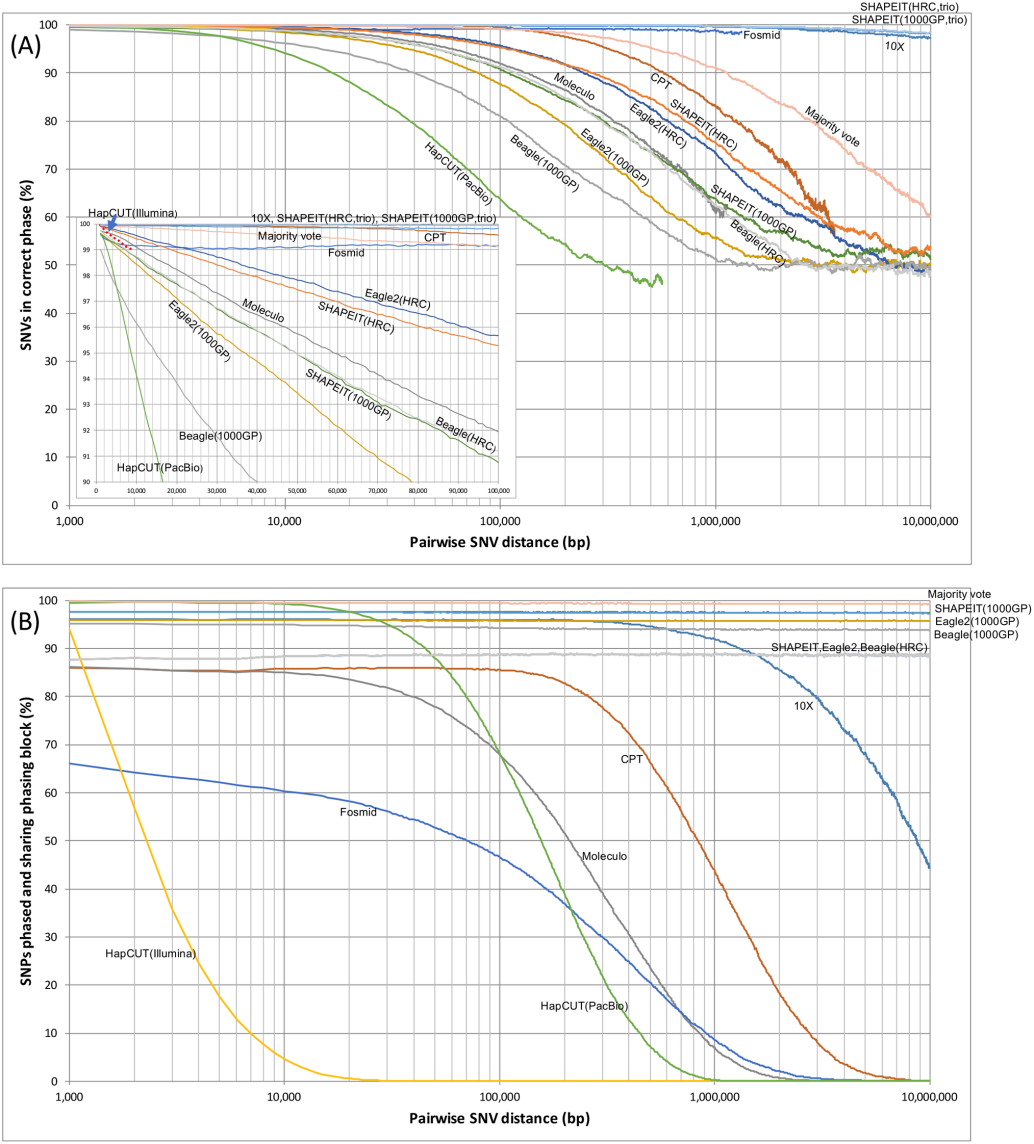
Phasing performance comparison based on pairwise SNV haplotype assignment. (A) Phasing accuracy. Probability that a pair of SNVs on the same phasing block is correctly phased with respect to each other as a function of the distance between the pair. (B) Phasing yield. Probability that a pair of SNVs are phased in the same phasing block as a function of the distance between the pair.

We measured phasing accuracy and yield measures across pairs of SNVs as in the study by Snyder et al. [8] (Fig 3). As shown in Fig 3A, the pairwise SNV phasing accuracy of the 10X, CPT-seq, and fosmid sequencing strategies stayed above 99% (i.e. 1 error out of 100 heterozygous sites) for distances of >100 kb between SNV pairs. At 99% phasing accuracy, the majority vote approach was up to 124 kb. Furthermore, if an even higher phasing accuracy of 99.9% was considered, the 10X method achieved a distance of 362 kb. For the strategy leveraging SHAPEIT along with the HRC panel and the PacBio reads, the same level of phasing accuracy (99.9%) was obtained for distances up to ~7 kb. Importantly, a tremendous improvement was observed with the SHAPEIT approach with the inclusion of parental genotype and using the HRC panel as background, which produced a 99.9% phasing accuracy for much longer distances up to ~429 kb (Fig 3A). Fig 3B depicts the phasing yield and represents the probability that a pair of SNVs are phased in the same phasing block as a function of the distance between the pair. Considering a 100 kb phasing yield distance, most lab-based methods are at or below 86% phasing yield including Moleculo, CPT, and fosmid, whereas 10X, Beagle(1000GP), Eagle2(1000GP), SHAPEIT(1000GP), and also majority voting are above 94% phasing yield.

#### Proportion of fully phase-resolved genes

In fully phased genes, haplotype blocks span the entire gene lengths, thus providing critical information on gene-based haplotypes. Such information could be especially useful for recessive disease variant analyses. Despite the large range of haplotype QAN50s generated by the different phasing approaches, a high proportion of fully phased genes (i.e., all heterozygous SNVs within a gene were correctly phased) was produced across laboratory- and population-based phasing methods including 10X and SHAPEIT approaches with the 1000GP panel and parental genotype at 70% or higher. The remaining SHAPEIT approaches using the 1000GP panel (reference panel alone or with read data) achieved 61 to 68%. This was followed by HapCUT using PacBio reads and Eagle2 based on the 1000GP panel, in which about 52% of genes were completely phased (Fig 1A, Tables 1 and 2, and S1 Fig). As described above the majority voting approach produced 89.1% fully phased genes.

The number of incorrectly phased or unphased sites for a group of disease-associated genes with extensive gene spans from 15 to 305 kb including *MEFV*, *DHODH*, *MYPN*, *APC*, *CFTR*, and *TTN* are shown in Table 3. For this gene set, using metrics that measures the total number of incorrectly phased or unphased sites from the 6 genes, among the best performing approaches are the majority vote approach (1 erroneous site), followed by 10X (4 sites) and SHAPEIT approaches (6 sites).

**Table 3:** Phasing accuracy of disease-associated genes for the reference individual NA12878.

Gene Symbol				MEFV	DHODH	MYPN	APC	CFTR	TTN	Total
Gene ID				ENSG00000103313	ENSG00000102967	ENSG00000138347	ENSG00000134982	ENSG00000001626	ENSG00000155657	
Disease involved				Mediterranean fever	Miller syndrome	Cardiomyopathy	Familial adenomatous polyposis 1	Cystic fibrosis	Cardiomyopathy	
Length (bp)				14,600	16,468	105,863	138,742	250,188	304,814	
# Heterozygous SNPs				25	16	172	88	5	56	
Method	Reference panel used	Read data used	Parental genotype used							
10X	-	-	-	0	0	1	0	1	2	4
CPT	-	-	-	1	0	7	2	5	12	27
Moleculo	-	-	-	2	0	8	2	3	3	18
Fosmid	-	-	-	7	5	40	8	0	2	62
Eagle2	1000GP	-	-	1	0	1	6	0	4	12
Eagle2	HRC	-	-	0	0	3	1	0	4	8
SHAPEIT	1000GP	-	-	0	0	5	5	0	2	12
SHAPEIT	HRC	-	-	0	0	3	1	0	4	8
Beagle	1000GP	-	-	1	0	5	6	0	2	14
Beagle	HRC	-	-	0	0	4	1	0	4	9
HapCUT	-	Illumina DNA	-	6	8	18	43	5	29	109
HapCUT	-	PacBio DNA	-	0	0	3	0	2	6	11
SHAPEIT	1000GP	-	Yes	0	0	1	5	0	0	6
SHAPEIT	1000GP	Illumina DNA	-	0	0	1	5	0	4	10
SHAPEIT	1000GP	Illumina DNA	Yes	0	0	1	5	0	0	6
SHAPEIT	1000GP	Illumina RNA-Seq	-	0	0	3	5	0	4	12
SHAPEIT	1000GP	Illumina DNA and RNA-Seq	-	0	0	1	5	0	4	10
SHAPEIT	1000GP	PacBio DNA		0	0	1	5	0	0	6
SHAPEIT	1000GP	PacBio DNA	Yes	0	0	1	5	0	0	6
SHAPEIT	HRC	-	Yes	0	0	3	1	0	4	8
SHAPEIT	HRC	Illumina DNA	-	0	0	3	1	0	4	8
SHAPEIT	HRC	Illumina DNA	Yes	0	0	3	1	0	4	8
SHAPEIT	HRC	PacBio DNA	-	0	0	3	1	0	4	8
SHAPEIT	HRC	PacBio DNA	Yes	0	0	3	1	0	4	8
Consensus by majority vote	-	-	-	0	0	1	0	0	0	1

The number of incorrectly phased or unphased sites are shown for each phasing approach.

#### Properties of switch errors

As noted, switch errors occur when a variant location is incorrectly phased with respect to its neighboring variants. Among the phasing approaches tested, as shown in Table 4, the number of switch errors ranged from 1,050 to 25,077. To further understand the distribution of switch errors, we asked whether the same switch error sites are observed across phasing methods, and whether certain genomic regions are more prone to switch errors. A pairwise comparison of switch errors based on genomic positions is shown in Table 4. Overall, population-based phasing approaches (i.e. all the strategies involving SHAPEIT, whether leveraging WGS reads or not, as well as Beagle and Eagle2) shared the highest proportion of switch error sites in common. This is probably related to the fact that these approaches all rely on a reference panel to provide statistical support for resolving phase. Note that the majority voting approach reduced the number and proportion of switch errors that were commonly shared among the population approaches, which highlights the advantage of combining multiple phasing prediction.

**Table 4:** Comparing the genomic location of switch errors across phasing approaches.

	10X	CPT	Moleculo	Fosmid	Eagle2 (1000GP)	Eagle2 (HRC)	SHAPEIT (1000GP)	SHAPEIT (1000GP, Illumina)	SHAPEIT (1000GP, PacBio)	SHAPEIT (HRC)	SHAPEIT (HRC, Illumina)	SHAPEIT (HRC, PacBio)	Beagle (1000GP)	Beagle (HRC)	HapCUT (Illimina)	HapCUT (PacBio)	Consensus (majority vote)
10X		0.01	0.00	0.00	0.00	0.00	0.00	0.01	0.01	0.00	0.00	0.01	0.00	0.00	0.01	0.00	***0*.*17***
CPT	23		0.01	0.00	0.01	0.01	0.01	0.01	0.02	0.01	0.01	0.01	0.01	0.01	0.00	0.01	0.02
Moleculo	25	81		0.00	0.02	0.01	0.02	0.02	0.03	0.02	0.02	0.02	0.02	0.02	0.00	0.01	0.01
Fosmid	11	17	47		0.00	0.00	0.01	0.01	0.00	0.00	0.00	0.00	0.00	0.00	0.00	0.00	0.01
Eagle2 (1000GP)	46	195	393	95		***0*.*09***	***0*.*28***	***0*.*22***	***0*.*13***	***0*.*09***	***0*.*07***	***0*.*05***	***0*.*07***	***0*.*07***	0.00	0.02	0.02
Eagle2 (HRC)	18	58	154	24	***1*,*421***		***0*.*07***	***0*.*08***	***0*.*08***	***0*.*20***	***0*.*19***	***0*.*15***	0.04	***0*.*13***	0.00	0.00	***0*.*09***
SHAPEIT (1000GP)	64	244	***525***	115	***6*,*804***	***1*,*273***		***0*.*40***	***0*.*20***	***0*.*11***	***0*.*08***	***0*.*05***	***0*.*07***	***0*.*06***	0.00	0.01	0.02
SHAPEIT (1000GP, Illumina)	66	207	472	91	***4*,*678***	***1*,*050***	***8*,*311***		***0*.*24***	***0*.*11***	***0*.*11***	***0*.*07***	***0*.*07***	***0*.*07***	0.00	0.01	0.02
SHAPEIT (1000GP, PacBio)	42	152	347	35	***2*,*406***	***650***	***3*,*827***	***3*,*472***		***0*.*11***	***0*.*11***	***0*.*12***	***0*.*05***	***0*.*06***	0.00	0.02	0.03
SHAPEIT (HRC)	20	76	249	34	***1*,*553***	***1*,*262***	***2*,*116***	***1*,*671***	***1*,*037***		***0*.*34***	***0*.*23***	0.05	***0*.*12***	0.00	0.01	***0*.*06***
SHAPEIT (HRC, Illumina)	21	69	211	30	***1*,*228***	***1*,*007***	***1*,*568***	***1*,*517***	***907***	***2*,*077***		***0*.*26***	0.04	***0*.*11***	0.00	0.01	***0*.*06***
SHAPEIT (HRC, PacBio)	20	58	166	20	***828***	***697***	***985***	***895***	***857***	***1*,*290***	***1*,*158***		0.03	***0*.*08***	0.00	0.01	***0*.*06***
Beagle (1000GP)	45	177	***510***	101	***2*,*637***	***998***	***2*,*719***	***2*,*276***	***1*,*510***	***1*,*357***	***1*,*071***	***779***		***0*.*05***	0.00	0.02	0.01
Beagle (HRC)	36	76	253	61	***1*,*447***	***1*,*228***	***1*,*472***	***1*,*218***	***795***	***1*,*305***	***1*,*083***	***726***	***1*,*571***		0.00	0.01	0.04
HapCUT (Illimina)	23	8	16	1	36	10	24	21	18	5	5	6	45	15		0.01	***0*.*09***
HapCUT (PacBio)	85	116	243	96	***500***	89	***503***	421	444	150	140	153	***643***	227	161		0.01
Consensus (majority vote)	358	69	134	42	303	364	298	290	215	348	267	202	240	342	217	172	

Below the diagonal: the number of common errors between a pair of approaches are shown (500 or more shown in bold). Above the diagonal: Jaccard index calculated as the number of common errors divided by the size of the union of the errors are shown (0.05 or higher shown in bold).

**Fig 4:**
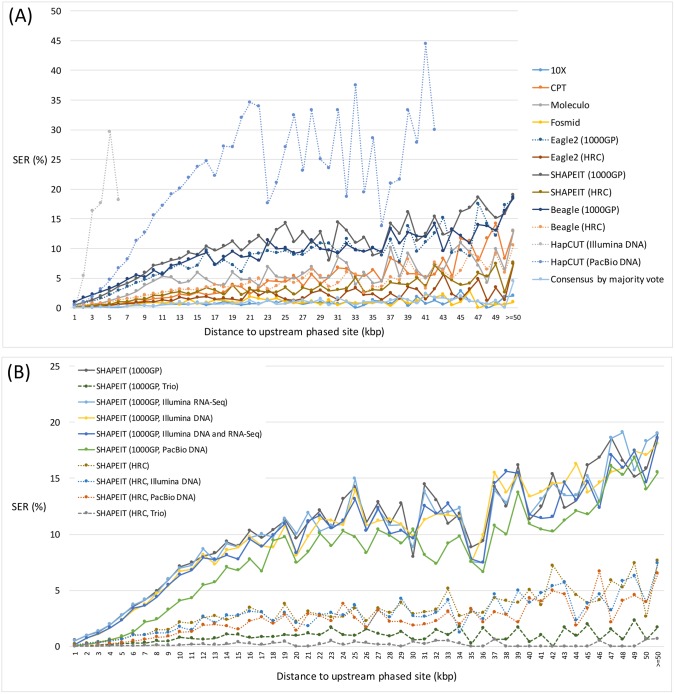
Phasing accuracy and SNV density. (A) Basic phasing approaches. (B) SHAPEIT phasing supplemented with reference panel, sequence read, or parental genotype information. Switch error rates for various phasing strategies are shown as a function of distance between a heterozygous site and its upstream phased site.

**Fig 5:**
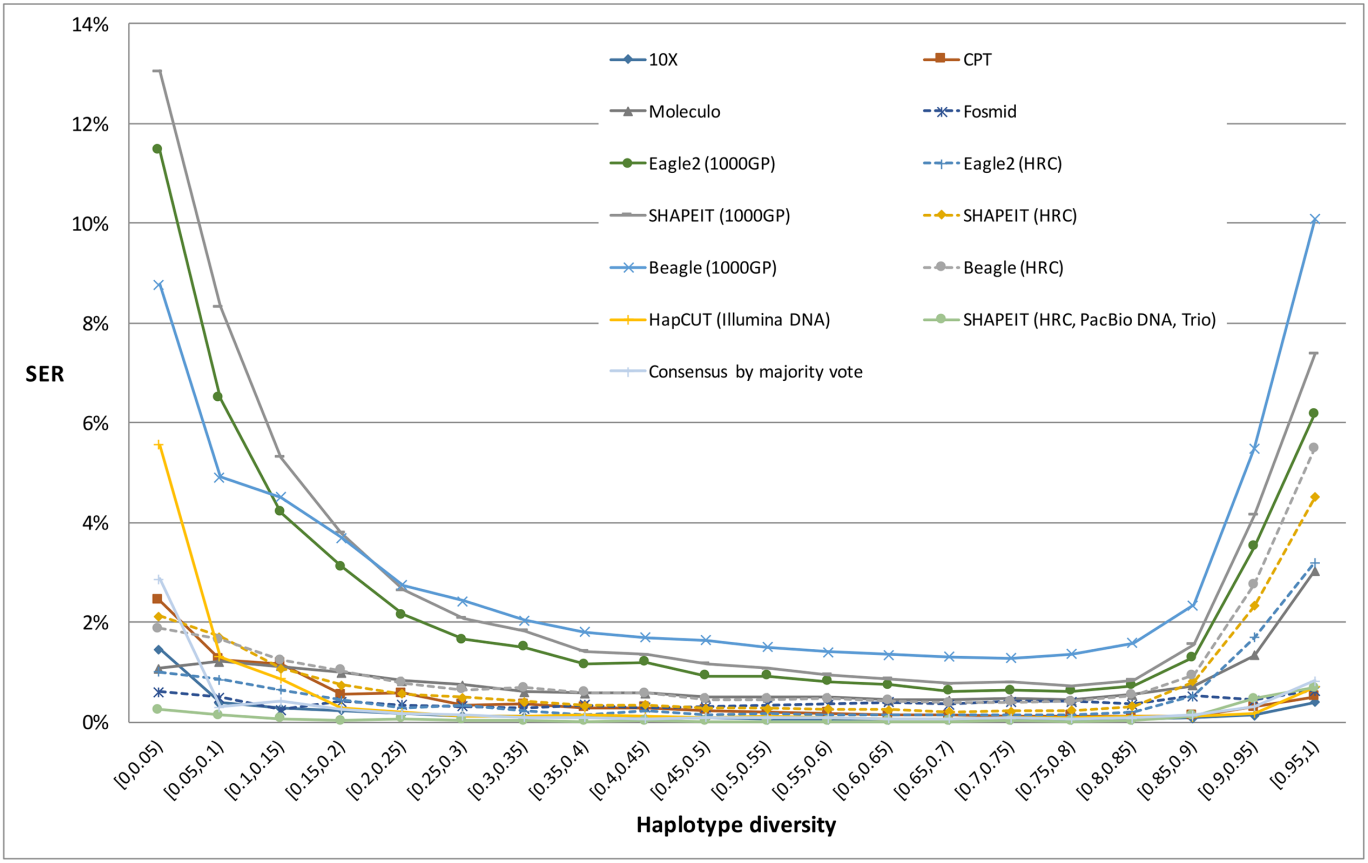
Phasing accuracy and haplotype diversity. Switch error rates for various strategies are shown as a function of haplotype diversity of a reference population based on the 1000GP reference panel.

Next, we assessed the specific DNA sequence context, including SNV density, haplotype diversity and repeat regions such as LINE, SINE, LTR and others, and their potential correlation with switch errors. SNV density was assessed indirectly by measuring the distance between a given heterozygous site and its immediate upstream phased heterozygous site. In all approaches tested, the switch error rate rises as the distance to upstream site increases, and the increase in error rate is more dramatic for strategies leveraging the HapCUT algorithm (Fig 4A). This may be due to the fact that unlike population-based phasing approaches, which make use of reference haplotypes, HapCUT only relies on the physical linkage information provided by sequencing reads. For SHAPEIT population-based approaches, using a larger reference panel (i.e. the HRC panel instead of 1000GP panel) or parental genotype helped reduce error rate for long distances (Fig 4B). Fig 5 shows that variations in haplotype diversity (derived from 1000GP data) are also associated with switch errors. Specifically, regions with a low haplotype diversity have a higher number of switch errors. This is consistent with the assumption that regions with low haplotype diversity usually carry a smaller number of SNVs and therefore a low SNV density. Finally, we also showed that switch errors are in general consistently distributed across the LINE, SINE, and LTR repeat regions (S3 Table).

### The effects of reference panel composition and size

**Fig 6:**
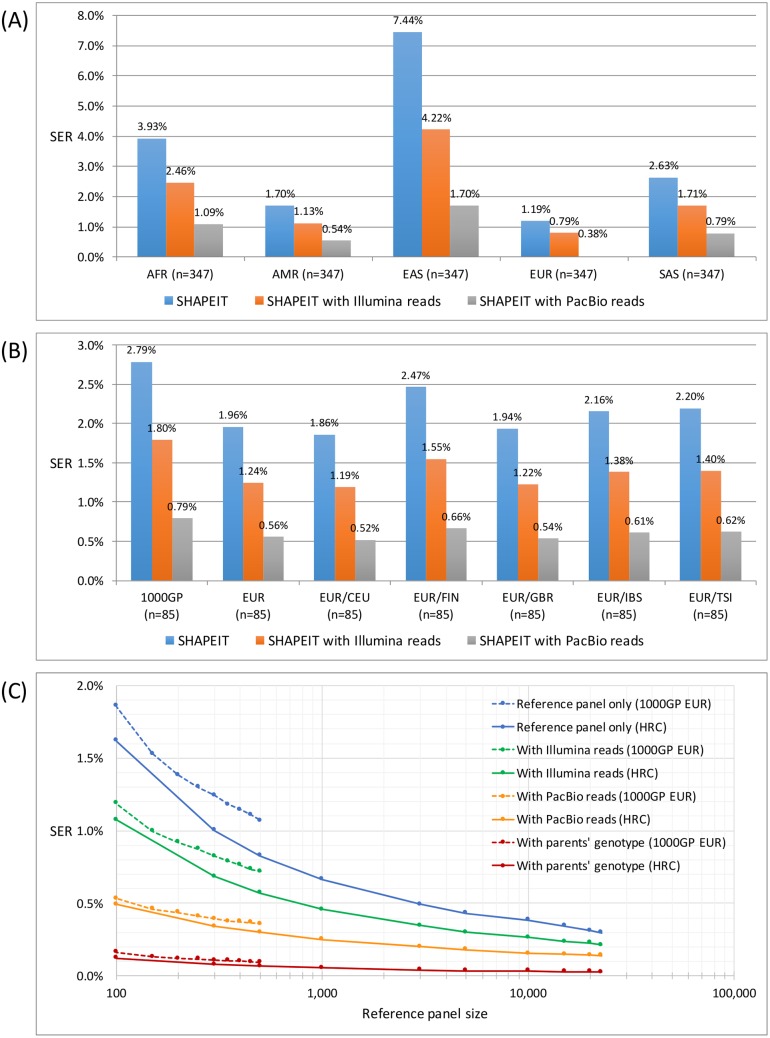
Phasing accuracy of SHAPEIT approaches and the choice of reference panels used. (a) Effect of population supergroups on phasing accuracy. Five supergroups of the same size (n = 347) were collected from the 1000GP and used as the reference panel for SHAPEIT (no read) phasing, or together with Illumina or PacBio reads for the NA12878 individual. The best SER was achieved by EUR, to which the NA12878 individual belongs. (b) Effect of population subgroups on phasing accuracy. Population subgroups of the same size (n = 85) were collected from the 1000GP, EUR, and each of five subpopulations in EUR and used as the reference panel for SHAPEIT phasing of the NA12878 individual. No major improvement on SER was observed among EUR and its 5 subgroups including EUR/CEU to which the individual NA12878 belongs. (c) Effect on phasing accuracy as SER as a function of reference panel size, compared with the inclusion of WGS reads or familial information from parental genotype. Reference panels containing up to 502 individuals from the 1000GP EUR group or 23k individuals from HRC were used as the population background for SHAPEIT phasing of the NA12878 individual.

To determine what, if any, impact the composition of a reference panel had on the strategies relying on population-based approaches, we examined the effects of the ancestral origins and size of the reference panel using SHAPEIT. The SERs were measured using the same number of individuals (n = 347) from each of well-known population ‘supergroups’ associated with the 1000GP data, namely AFR, AMR, EAS, EUR, and SAS, for the assessments of the SHAPEIT-based strategies. The EUR supergroup, when used as the reference panel alone, produced the lowest switch error rate (Fig 6A).

We further investigated the effect of ancestral subpopulations on phasing accuracy, by dividing the EUR supergroup into well-known 1000GP subpopulations: CEU, FIN, GBR, IBS, and TSI, with each group including the same number of individuals (n = 85). We compared the phasing accuracy of the subpopulations against the supergroup EUR and the overall 1000GP mixed population at the same group size (Fig 6B). The SHAPEIT switch error rates obtained for CEU, EUR, and 1000GP were 1.9, 2.0, and 2.8%, respectively. Given that the reference genome NA12878 belongs to the EUR population and the CEU subgroup, our results suggest that a reference panel that contains matching ancestry with the query genome at the supergroup (i.e. EUR) level, and not necessarily at the subpopulation level (i.e. CEU), was sufficient to achieve excellent phasing performance.

We also examined the effect of reference panel size by randomly subsampling from each of the 1000GP EUR (n = 502) and HRC (n = 22,690) panels. For both panels, the phasing accuracy was improved by increasing the reference panel size, and the improvement diminished as the panel size increases in all SHAPEIT approaches with and without WGS reads or parents’ genotype data. Specifically, when 50X PacBio reads were supplied, only ~500 HRC individuals were required to obtain the same accuracy as achievable by using the entire 23k HRC reference panel alone (i.e. ~0.3% SER). Furthermore, when familial information from parental genotype data was used, the SER obtained was 0.1% with 100 HRC individuals and as low as 0.03% when using the entire panel of 23k HRC individuals (Fig 6C).

## Discussion

Current human WGS strategies do not provide phase information by default. This creates issues for identifying certain clinically-meaningful phenomena, such as compound heterozygosity, as well as population-level phenomena such as haplotype diversity and LD patterns that could help resolve migratory patterns and mutation origins. We evaluated 11 different available strategies to resolve phase for human genomes. In general, these strategies fell into 3 different categories: those that rely purely on building haplotypic contigs from DNA sequencing reads or unique laboratory-based approaches through genome dilution, such as the 10X, CPT, fosmid pools, and Moleculo; those that rely on purely population-based strategies in which a reference panel of previously resolved haplotypes is leveraged to probabilistically assign the most likely haplotype pair to an individual based on their genotype data (i.e. Beagle, Eagle2, SHAPEIT); and those that rely on some combination of sequence read- and population-based constructs, and/or parent offspring trios genotype information. Our results suggest that: (1) the recently developed large comprehensive reference panel by the HRC greatly enhance phasing performance; and (2) population-based phasing performance can be greatly enhanced with family trio data that can match or surpass the precision of laboratory-based methods. In all, laboratory-based methods are more expensive (e.g., $2,000 per genome using the 10X Genomics approach [40]), and often require protocols that add complexity to workflows needed to use them but are likely to come down in costs. For cost-effectiveness, a combination of computational methods and input data types as shown in this study likely worth considerations.

In our view, by far the most efficient and least expensive current approaches to phasing are likely going to be based on computational methods leveraging large reference panels such as the HRC. Our analyses showed that population-based phasing performance can be improved if WGS/RNA read data are available. Long read-based strategies (e.g. PacBio) provide an additional advantage over other sequencing strategies for their ability to link kilobase-range long distance variants. However, the best improvement to population phasing performance, which involved a 10-fold reduction of errors and also a 10-fold increase in haplotype block lengths, was the addition of parental genotype data. We also proposed a majority voting approach to exploit the possibility of combining phasing strategies to improve the overall SNV site coverage and the number of phase-resolved genes, and also to build consensus from multiple predicted haplotypes to simplify downstream analysis. Traditional strategies to phasing genomes that involve sequencing the parents (or relatives) or a target individual are enjoying a resurgence because of that strategy’s ability to also help validate *de novo* or private mutations in clinical contexts. Although such a strategy appears costly in terms of the amount of sequencing that needs to be performed as it also depends on the availability of relatives, the continuous reduction in sequencing cost could favor the familial approach for improving the performance of computational phasing methods.

With regard to the fosmid pool sequencing dataset, for comparison with the NA12878 GIAB gold standard and other phasing approaches we used the liftOver tool [26] to convert the genomic coordinates from the NA12878 fosmid study from NCBI36 (hg18) to GRCh37 (hg19). As the liftover process for NA12878 fosmid data may have led to loss of some haplotype blocks, the fosmid-based phasing result for the European individual ‘‘Max Planck One” (MP1) from a previous study is mentioned here for additional comparison. For the NA12878 individual, approximately 0.48 million fosmid clones were sequenced (i.e. 32 pools with 15,000 clones per pool), which was estimated to provide 5X coverage of the genome with 77% of SNVs phased [10]. For the MP1 individual, deep sequencing of approximately 1.44 million fosmid clones (96 pools) equivalent to 14X coverage of the genome had led to 99% SNVs and 81% genes phased [10].

We focused on phasing performance for the autosomal chromosomes. Because chromosome X has a number of specific features, such as variants within it exhibiting unique inheritance patterns, it does contain genomic features, including the pseudo-autosomal regions (PAR) and non-PAR segments, that would require special data handling and analysis modifications to be accommodated in our analyses. For NA12878, who is a female, we attempted a phasing comparison for the non-PAR region of chromosome X. The SER obtained with 10X and Moleculo was 0.15 and 0.60% respectively, which was about 2.43 and 1.19 times higher than the autosomes. For chromosome X, SHAPEIT phasing produced haplotype blocks with SER at 0.3%. The addition of parental genotype reduced the SER to 0.05% (S4 Table).

Our analyses do raise some important questions. First, the accuracy of our results relies on the NIST-GIAB gold standard variants used in this study. Recently, a further improved high confidence NA12878 variant dataset (Platinum genotype calls) was developed and validated using extensive family information spanning 3 generations and 17 members, and incorporating 6 different variant calling pipelines [41]. For the NA12878 individual, a total of 3.5 million SNVs (heterozygous or homozygous) were identified in the Platinum variant dataset containing 800,564 additional SNVs not included in the NIST-GIAB set, and conversely a set of 62,946 SNVs in the NIST-GIAB set are not included in the Platinum dataset [41]. Nevertheless, over 97.7% SNVs of the NIST-GIAB variant set used in this study overlapped with this high confidence variant dataset and have high genotype concordance level (>99.99%) [41]. Using the Platinum dataset [41] as a gold standard, we observed similar phasing performance for the different approaches tested (S3 Fig). Second, we obtained phased genomes deposited in the public domain by, e.g. 10X and Moleculo, and these were likely put together by the relevant research teams to showcase these technologies. Thus, the results of these strategies may have been pursued with some level of optimization that is not likely to be obtained with routine and large-scale use of these strategies.

Finally, our analyses focused on SNVs and did not consider short insertion-deletion polymorphisms (indels) and structural variants such as moderate to large indels, copy number variants (CNVs), inversions, or other forms of structural variations (SVs). Such variations have a significant role to play in many diseases and are hence very important to consider. In addition, certain combinations of SVs and CNVs might be very important to resolve for population-based studies. For example, variants within different copies of a gene may help resolve the origins and recent evolution of those copies. Ultimately, since humans are indeed a diploid species, the ability to phase human genomes using fast and low-cost computational approaches will push diploid genome analysis to a new level.

## Supporting information

S1 Table SNV genotype of NA12878 and the parents in chromosomes 1–22. Based on the NA12878 parent offspring family trio genotype data, the total number unphasable sites by conventional Mendelian segregation rules were estimated to include 360,241 triple heterozygous sites and 463,995 sites with a heterozygous variant in NA12878 but no variant called (homozygous reference or undetermined genotypes) in both parents. Thus, at best only 67% (824,236 out of 2,467,785) of NA12878 heterozygous SNV sites can be phase-resolved by the conventional transmission approach.
(XLSX)

S2 Table Switch error rate and percentage of phased SNVs breakdown by chromosome. (XLSX)

S3 Table Switch errors across the LINE, SINE, and LTR repeat regions. (XLSX)

S4 Table Switch error rate of 10X, Moleculo, and various SHAPEIT phasing approaches for X chromosome. (XLSX)

S1 Fig Performance of all phasing methods compared in this study ranked by individual metrics: (a) Switch error rate, (b) Haplotype block size (QAN50), and (c) Percentage of fully phased genes. (TIF)

S2 Fig Performance of majority vote phasing compared to 8 individual phasing methods ranked by individual metrics: Switch error rate, % phased SNVs, and % fully phased genes. (TIF)

S3 Fig Comparison of phasing performance using two different datasets as the gold standard. A total of 1,697,789 and 2,084,089 phase-resolved heterozygous SNVs on 22 autosomes were contained in the NIST-GIAB and the Platinum datasets, respectively.
(TIF)
