# Supplementary figures and images for: Comparison of phasing strategies for whole human genomes
Source: PLoS Genet. 2018 Apr 5;14(4):e1007308. doi: 10.1371/journal.pgen.1007308 (PMC5903673; doi:10.1371/journal.pgen.1007308)

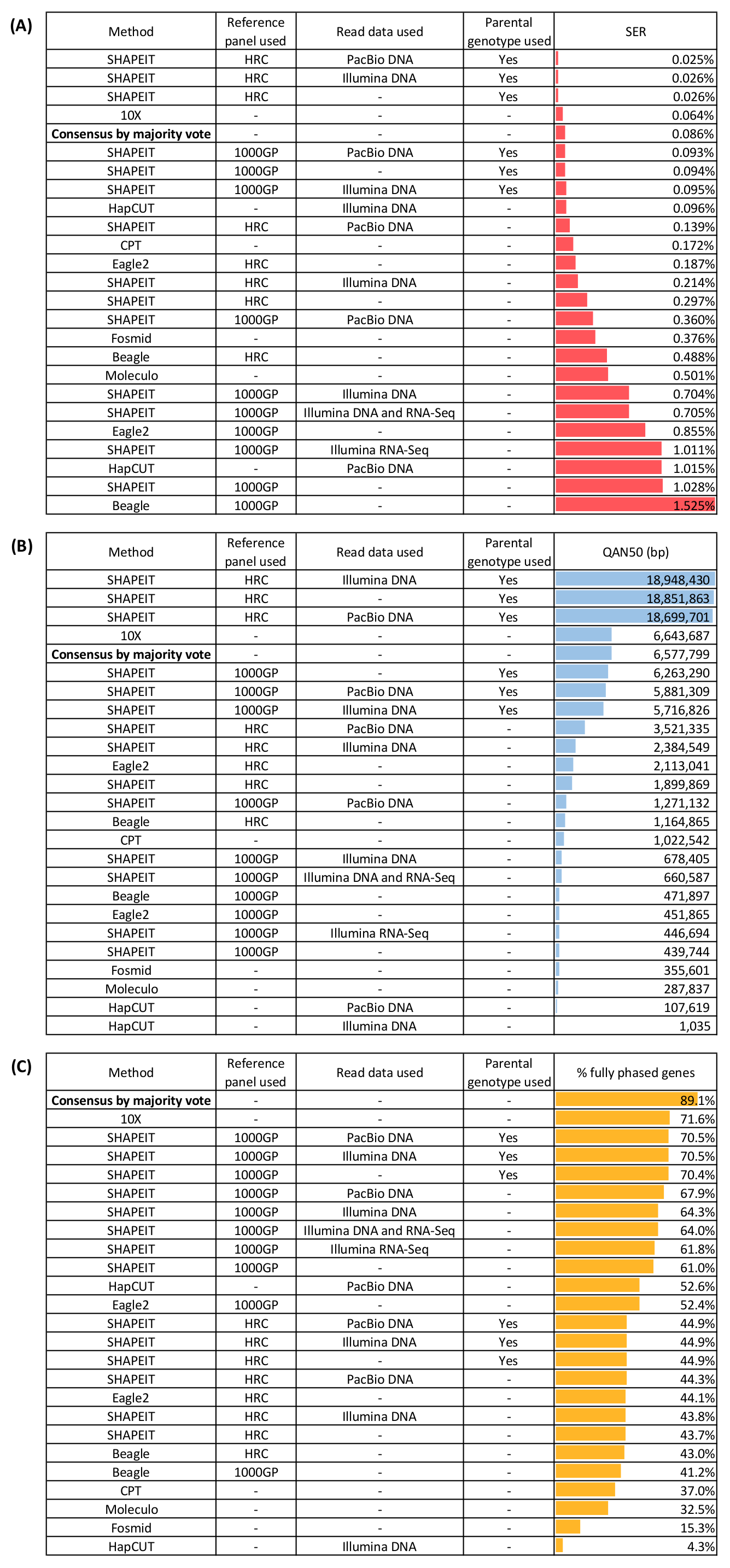

Supplement: S1 Fig — (TIF) [file pgen.1007308.s005.tif]

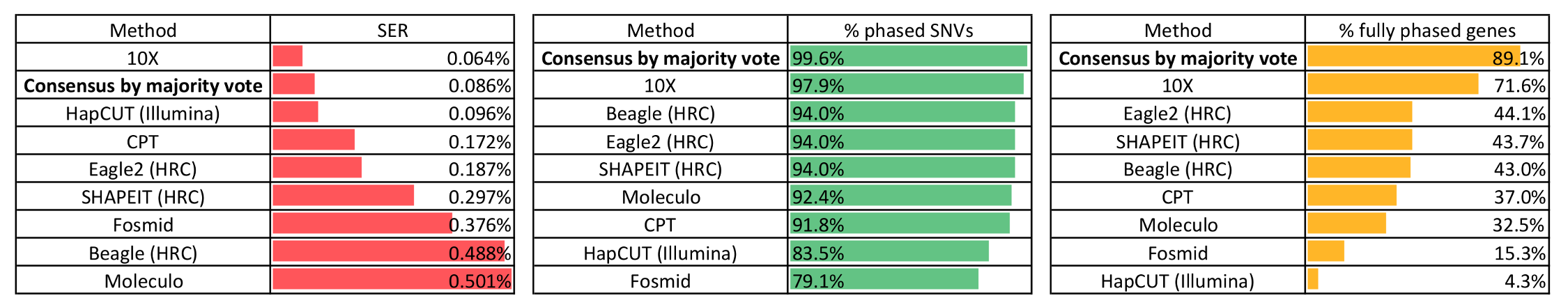

Supplement: S2 Fig — (TIF) [file pgen.1007308.s006.tif]

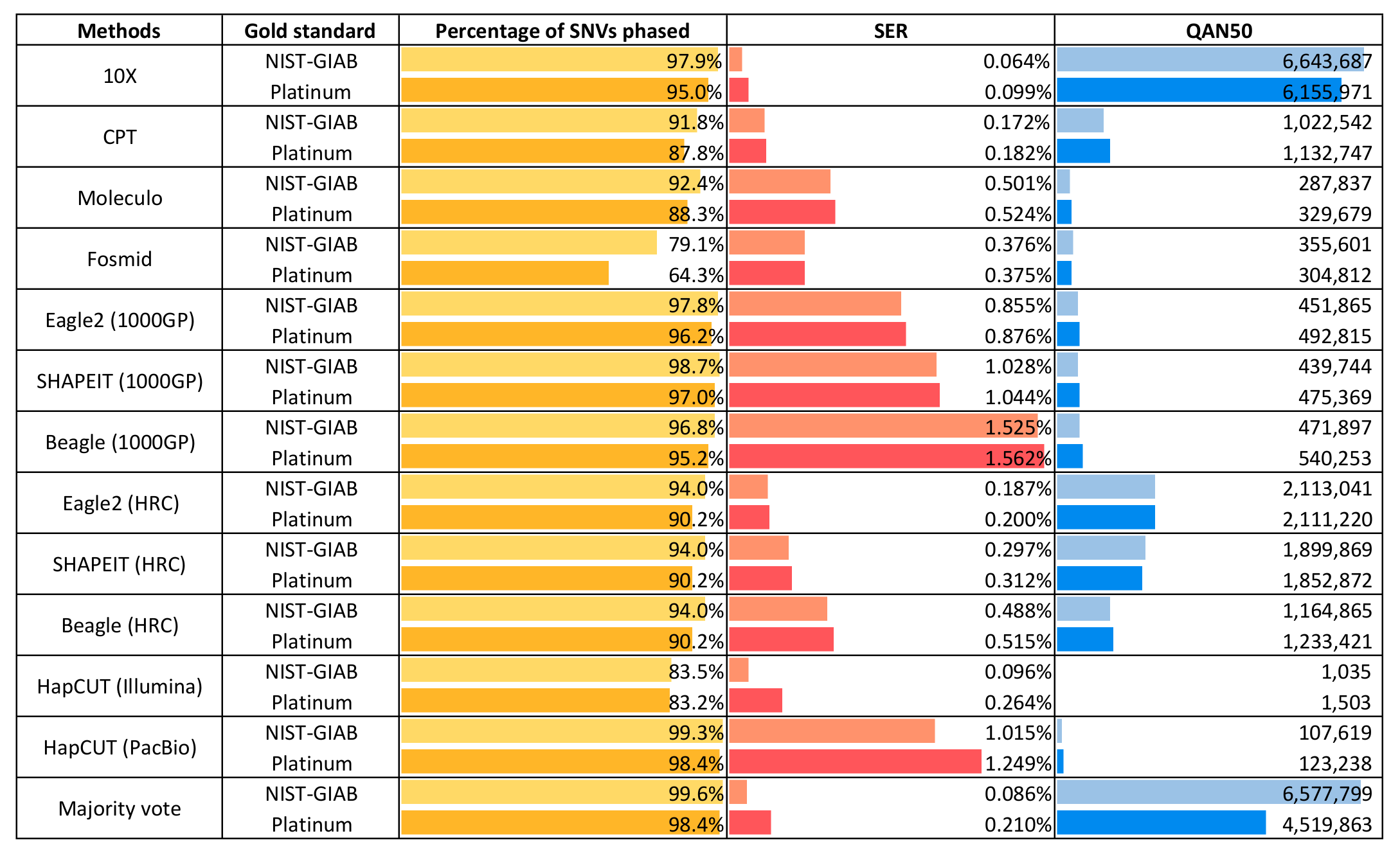

Supplement: S3 Fig — A total of 1,697,789 and 2,084,089 phase-resolved heterozygous SNVs on 22 autosomes were contained in the NIST-GIAB and the Platinum datasets, respectively. (TIF) [file pgen.1007308.s007.tif]
